# OccIDEAS: Retrospective Occupational Exposure Assessment in Community-Based Studies Made Easier

**DOI:** 10.1155/2009/957023

**Published:** 2009-10-15

**Authors:** Lin Fritschi, Melissa C. Friesen, Deborah Glass, Geza Benke, Jennifer Girschik, Troy Sadkowsky

**Affiliations:** ^1^Western Australian Institute for Medical Research, University of Western Australia, Perth, Western Australia 6012, Australia; ^2^Environmental Health Sciences, School of Public Health, University of California, Berkeley, CA 94720-7360, USA; ^3^Monash Centre for Occupational and Environmental Health, Department of Epidemiology and Preventive Medicine, Monash University, Melbourne, Victoria 3004, Australia; ^4^Data Scientists Pty Ltd, Sunshine Coast, Queensland 4560, Australia

## Abstract

Assessing occupational exposure in retrospective community-based case-control studies is difficult as measured exposure data are very seldom available. The expert assessment method is considered the most accurate way to attribute exposure but it is a time consuming and expensive process and may be seen as subjective, nonreproducible, and nontransparent. In this paper, we describe these problems and outline our solutions as operationalized in a web-based software application (OccIDEAS). The novel aspects of OccIDEAS are combining all steps in the assessment into one software package; enmeshing the process of assessment into the development of questionnaires; selecting the exposure(s) of interest; specifying rules for exposure assignment; allowing manual or automatic assessments; ensuring that circumstances in which exposure is possible for an individual are highlighted for review; providing reports to ensure consistency of assessment. Development of this application has the potential to make high-quality occupational assessment more efficient and accessible for epidemiological studies.

## 1. Introduction

As we spend a quarter of our lives at work, occupational risk factors for various health conditions are an important area of study. However, assessing occupational exposure, particularly within community-based studies such as case-control studies or population-based cohort studies, remains a substantial challenge. To address this challenge, community-based studies have employed several methods to assess exposure [1, 2]—exposure measurements, self-report, job exposure matrices, and expert assessment.

Exposure measurements are considered the “gold standard” in cohort studies based within a single industry [3]. But in community studies the process of retrieving any exposure measurements would require individually contacting each employer from each subjects' past jobs. Given the many employers for each subject, and the time which may have passed since the job, doing so is not practicable in most large studies [2]. For example, the 1479 subjects in a recent case-control study of prostate cancer reported between 1 and 33 jobs each, with a mean of 8.4 jobs per person, the earliest job started in 1932 and there was a total of over 13 000 jobs [4]. In studies with a more confined scope, such as those just examining current job [5], or those in which a biomarker is available [6] it may be possible to measure exposures but usually only large companies will have historical measurements of occupational exposure.

Subjects have been asked to self-report exposures but their ability to do so accurately varies with the agent of interest. Workers have difficulty in assessing the extent or level of exposure because this requires making comparisons of exposure across different industries with which they usually have no familiarity [7]. There is also a risk of recall bias due to rumination by the subjects with the disease [1, 2, 8], although one small study investigating this found little supporting evidence for the existence of bias [9].

Generic job exposure matrices using job title alone assign the same exposure to all workers in the same job. This makes misclassification of exposure likely, particularly in jobs which have wide variability in the work undertaken (such as labourers, nurses, or laboratory workers) [10, 11].

One solution is to subdivide the jobs into more specific categories, and this is the basis of the expert assessment method [10] which might be thought of as an individualized job exposure matrix. In this method, a full job history is provided by the subjects. The subjects are then asked questions from job-specific modules (JSMs). JSMs are questionnaires which contain questions relevant to exposure determinants for tasks and processes done within a particular job (e.g., carpenter, driver, health professional). Finally, an expert reviews the interview responses and assigns exposures. The expert's assessment will change according to the answers given to the questions in the JSMs, meaning that this method can accommodate the substantial variability within jobs. Expert assessment usually outperforms self-reports as experts can augment their assessment with their experience, published literature, and where available, national exposure databases [10, 12]. In addition, experts are able to calibrate their assessments of levels across a wide range of industries [2, 10].

However, the expert assessment process is extremely labour intensive. For example, assessment of the 13 000 jobs in the prostate cancer study mentioned above [4] took over 1000 hours of expert time. In addition, the assessment process is a “black box” which makes it difficult to fully justify the assessments objectively or for outsiders to determine how the expert arrives at the final decision. Experts do try to calibrate their own assessments but it is difficult to ensure consistency over time and over many different jobs. On the other hand, if funds and appropriate experts are available (particularly if a panel of experts are available), it is possible to undertake a very rigorous assessment which may be justified for the reduction of misclassification.

While not perfect, expert assessment is considered to be best practice in community-based studies for occupational risk factors [2, 13]. We sought to use recent technological developments to make the process of expert assessment cheaper, quicker, transparent, more efficient, and more consistent. In this paper, we describe a web application which automates a part of the expert assessment system (Occupational Integrated Database Exposure Assessment System, OccIDEAS).

## 2. Description of OccIDEAS

OccIDEAS is a web application written in Java which links the steps of the expert assessment system and automates some of the assessment steps. There are interfaces which allow users to do a range of tasks such as: develop new JSMs or edit existing ones; change the if/then rules in the JSMs; manage job history data; undertake interviews; view data and automatic assessments; and manually assess exposures.

When developing a JSM for a particular industry, a researcher needs to investigate the industry, the job, and the tasks within the job as well as the main agent exposures. In order to do this, the person who is developing the questionnaire reviews the literature, talks with experts, and collects questionnaires developed for the previous studies (particularly from [3, 10]). For each JSM there is an associated online collaborative discussion board which contains references used in creating the JSM and the rationale behind decisions to include questions or what level of exposure to assign.

Within OccIDEAS, questions are tagged with the exposure agents relevant to that question. An important design philosophy was to keep questions narrowly focussed to facilitate this tagging. So for example, instead of asking a question such as “What were other workers doing in the area where you were working?” a question might ask “Were you working in the area where metal was poured?” Tagging each question with its associated exposure agents allows automatic removal of a question from an interview if that agent is not of interest to the study, thus shortening the interview. For example, if the hypothesis of a study is that solvent exposure is the causative agent, only those questions relating to solvents would be retained, while ones tagged with other agents such as ionizing radiation or diesel exhaust would be dropped. In our prostate cancer study we based our questionnaires on questionnaires used in a study of non-Hodgkin lymphoma. Because of different hypotheses in the two studies we needed to remove questions relating to solvents and PCBs and add questions relating to oils, fertilizers, and exhaust fumes. This process previously took us several weeks of intensive reviewing and editing to modify the questionnaires. In OccIDEAS it takes less than half an hour as one simply selects the agents of interest and the template JSM is automatically modified to only include questions relating to those agents.

The assessment involves deciding on probability of exposure (none, possible, probable), level (none, low, medium, high), and frequency of exposure (weeks per year and hours per week). The tasks which result in probable exposure (and therefore the questions relating to those tasks) are usually clear, so that decision making rules can be assigned, for example, welding will result in probable UV exposure. The designation of “possible exposure” is used to highlight more difficult cases in which experts may need to examine the context of the job or free text answers in order to assign exposure, for example, not all welders are exposed to high levels of metal fume.

Generally, we define low level as above background but <10% TLV, medium as 10%–100% TLV, and high as >100% TLV at current TLV levels [14]. The option of “unknown level” is also available. For some agents such as shiftwork, physical activity, or sun exposure there is no TLV and the levels are related to a standard level. For example, shiftwork might be categorized as work over the graveyard (1 AM to 5 AM) shift (high exposure), work at night but not the graveyard shift (medium), and changing shifts but not involving night work (low). All levels used are recorded in the online documentation.

During the questionnaire development, the expert simultaneously develops exposure rules. These rules are if/then statements relating to particular answers to questions and provide an automatic exposure assessment. As a very simple example, in the JSM for forestry workers, questions and their answers in Table 1 lead to the automatic rules assigning wood dust exposure of “Probable” if the person chopped down trees with “High” level of exposure category if a chain saw was used and a “Medium” level of exposure if a hand saw was used.

Rules can also include information from the job history such as the country in which the job was done or the year of employment. Thus, for example, rules can specify that the exposure is “high” before 1983 and “low” afterwards. Level of exposure can be modified by the use of different types of personal protective equipment or ventilation.

During the data collection phase of a study, the job histories of the subjects are obtained and entered, either from a written questionnaire, or directly into the system by an interviewer or the subject. The study researchers or the interviewer then manually link the appropriate JSMs to each job using the title and main tasks as described by the subject. We explored the possibility of linking the JSMs to jobs automatically possibly using fuzzy logic, but the range of descriptions for jobs was found to be too broad to do this accurately.

The participant is then ready to have a computer-assisted interview, which may be done by an interviewer (in person or by phone) or online by the subjects themselves. Status reports can be used to track subjects who require interviews or are awaiting assessments. Interviewers can be trained to do the JSM assignment and to administer the JSM at the same time as the job history is taken; however if the subject enters their own job history, it is necessary to have a two-step process. Once the data collection for a subject is complete, the data from the job history and the answers to any JSMs are ready to be assessed for exposure.

The assessment of the probability, level, and frequency of exposure is performed on an agent-specific level. Assessments can be performed automatically by invoking the rules, or can be done manually. The invoking of the rules to produce an automatic assessment is controlled by the expert, who can run the rules for just one person or for a subset of subjects. The manual and automatic assessments are held separately so it is possible to compare independent assessments. Each triggered rule is displayed for the expert so that he or she can understand why a particular subject was assigned a specific exposure assessment. For each subject, the expert assessors can choose to assess the exposure independently, accept the automatic assessment, or modify the automatic assessment and provide comments on why they chose to do that. The comments are used to improve the template JSMs.

Given the low prevalence of occupational exposures in community-based studies, the rules are designed to be sensitive to possible exposure circumstances. If the exposure is very probable in a task, the rule will usually include a level of high, medium, or low. If there is less certainty whether the task involves exposure, or if the answer to a question is “do not know” then the automatic assessment would assign an “unknown” level. These cases would be priorities for the expert to review manually.

In population-based studies, the large number of jobs with no exposure result in a huge and unrewarding burden for the expert to review manually. In our prostate cancer study [4], 43% of the subjects had no exposure to any of the agents being assessed (metals, wood, oils, pesticides, fertilizers, exhaust fumes). However each of these unexposed subjects needed to be reviewed by the expert and we estimate this took about a quarter of the time, that is, over 250 hours. In OccIDEAS, all the subjects with no exposure can be reviewed easily in one report and batch confirmed as having no exposure (or individually assigned exposures if required). Since the prevalence of most agents in community-based studies is typically 1%–20% [10], this means that the expert can concentrate on examining the minority of jobs where exposure is likely rather than the large number of jobs with very low likelihood of any exposure. This represents a big time and hence cost saving for the exposure assessment process, reduces the repetitive nature of the work, and reduces the probability of misclassification. In addition, some agent/job combinations are likely to be less variable than others (e.g., in most nursing jobs an individual would be exposed to blood borne viruses, whereas only a few individuals would be exposed to ionizing radiation). The expert can therefore rapidly accept some of the auto assessments from the consistent combinations and spend their time on the more variable, difficult, and interesting assessments. This is less likely to result in expert assessor “burn out.”

## 3. Discussion

OccIDEAS is a new tool to manage the entire process of assessing occupational exposure in community-based studies. OccIDEAS has a wide range of features that allow experienced occupational epidemiologists to improve consistency and efficiency of their well-established processes. For epidemiologists without particular occupational expertise, OccIDEAS offers ready made questionnaire templates and the options for automatic rule assignments. Although such researchers would always need to consult with occupational exposure experts, the time involved in design of questionnaires and assessment of exposures is much reduced, meaning that assessing occupational exposures in population-based studies is possible for a wider range of epidemiological teams.

The huge range of jobs, time periods, and exposures within the scope of community-based studies makes it difficult to validate the expert exposure assessments, especially as the most relevant exposures of interest are usually those that occurred many years previously. Although the expert assessment approach has been used since 1981 [10], there are few validation studies available of the assessments. One study compared the ratings of a panel of occupational hygienists with measured data and found that the panel's specificity was high but sensitivity was variable [15]. A followup study using raters who were very experienced in the expert method found that the expert assessments had 90% sensitivity and were very accurate in their assessments of level and frequency of exposure [16]. Other studies have tried to validate just within certain industries [17]. We are currently undertaking a number of such limited validation studies.

To provide maximum benefits, considerable care and time are required in the initial design of questions and in the specification of rules. Once the template JSMs are established, however, the efficiency and quality gains are considerable. The rules do not eliminate the need for expert review and assessment but uses the experts' time more efficiently. By using the current pool of established OccIDEAS rules the highly-trained experts can spend their time on difficult and challenging assessments rather than on the simple non exposed jobs which are tedious to do and for which it is hard to maintain concentration.

The rules mean that the process of assessment is transparent and objective. The rationale behind each exposure decision is open for other researchers to examine and criticize. While this may seem threatening, this is one of the greatest strengths of OccIDEAS and it will lead to better assessments and therefore improved validity of study results. In addition, the questionnaire template system has the potential to improve consistency between studies. Occupational exposure assessment experts are not necessarily expert across the whole range of industries or whole range of possible chemical and physical exposures. OccIDEAS has the potential to pool and share expertise which has previously largely been confined to a single study or group of researchers.

Another advantage is in minimizing respondent burden and study costs while improving study data. The previous ways of minimizing the length of the interview have included asking JSMs only for those jobs held for long periods, or for only one of several similar jobs [4]. In OccIDEAS, only the information directly used in exposure assessment is asked in the JSMs, thus minimizing the time taken for the interview. This may mean that these short JSMSs can be used for more of the jobs in the person's job history. The compromise, however, is that it would be difficult to assess additional exposures after the data have been collected, although this is a limitation of most epidemiological studies.

OccIDEAS works behind the scenes using Java objects and a relational database management system. Thus it is possible to change the user interface so that questions are asked in different languages, while the objects behind the interface remain the same. We caution that the questions and rules would need careful revision if used in a country with occupational conditions very different to those in Australia and other Western countries. However the flexibility within the rule development functionality allows country of job to be specified in the rule. For example, the rule could say that if the country was China, exposure was high, whereas it might be medium in Australia.

OccIDEAS is open source software and a demonstration is available at http://www.occideas.org/. The existing JSMs (see Table 2) are available from a not-for-profit company which develops and provides the JSMs for a fee. All revenue are reinvested into expanding the number and quality of JSMs available as well as increasing the number of agents (Table 2) for which assessments can be performed. We welcome collaborators who can improve on existing JSMs and develop new ones. The process of developing new JSMs is never-ending. JSMs can continue to be developed for more and more uncommon jobs (e.g., art restorer, dental prosthetic technician). Combining international expertise will avoid duplication of effort and result in a general improvement in the quality of all studies.

OccIDEAS is a new tool for occupational exposure assessment in community-based studies which is only possible because of the increased computing capabilities available now. Our hope is that it can be used collaboratively to expand the quantity and improve the quality of occupational assessment in community-based studies.

## Figures and Tables

**Table 1:** Forestry worker JSM questions leading to exposure assessment rule for wood dust.

Question	Answer	Exposure rule
Did you chop down trees?	Yes	Probable exposure
→ How did you usually chop down trees?	Chainsaw	High level
	Hand saw	Medium level

**Table 2:** Currently available job specific modules and agents in OccIDEAS.

Job Specific Modules	Aluminium smelting industry
	Barber/hairdresser
	Carpenter/cabinet maker
	Driver/transport worker
	Dry cleaner
	Electrician/linesman
	Farmer
	Fishing/shipping/merchant seaman
	Flight attendant/frequent flyer
	Forestry and timbermill and lumber worker
	Gardener/groundskeeper
	Health professional
	Labourer
	Machinist
	Mechanic
	Military
	Miner
	Office worker
	Painter
	Petrol and gas station attendant
	Railway worker
	Shiftworker
	Teacher
	Tradesman
	Welder/boiler maker

Types of agents which can be assessed	Adhesives (solvent glues, water glues, heat glues, contact adhesives)
	Blood borne pathogens
	Combustion products (diesel exhaust, petrol exhaust, other exhausts, other PAHs)
	Fertilizers (mineral, natural)
	Formaldehyde
	Inorganic dusts (asbestos, fibreglass, silica, other inorganic fibres, other inorganic dusts)
	Metals (lead, other toxic metals, other metals)
	Nitrosamines
	Oils (natural, mineral, synthetic)
	Organic dusts (wood, grain, cotton, other organic dusts)
	PCBs
	Pesticides (organochlorines, organophosphates, phenoxy herbicides, other herbicides, other pesticides)
	Pigments (paints, dyes, other pigments)
	Radiation (ionizing radiation, UV, ELF, RF)
	Resins (acrylamide, resins)
	Shiftwork, jetlag
	Solvents (benzene, other aromatic solvents, chlorinated solvents, aliphatic solvents, alcohol)
	Sterilizing agents (ethylene oxide, other sterilizing agents)
